# LSTM-attention-guided graph neural networks for integrated genotype–Environment modeling in maize yield prediction

**DOI:** 10.1371/journal.pcbi.1013729

**Published:** 2026-05-20

**Authors:** Amir Morshedian, Mike Domaratzki

**Affiliations:** Department of Computer Science, University of Western Ontario, London, Ontario, Canada; University of North Texas, UNITED STATES OF AMERICA

## Abstract

This paper presents a deep-learning framework that combines an LSTM, a graph neural network (GNN), and transformer-style attention to model genotype–environment (G×E) effects for maize yield prediction. Weather data for a growing season is summarized using LSTM and encoded into a 21-dimensional embedding that is used as the environment node feature; 437,214 SNPs are summarized into 548 principal components that instantiate genotype nodes. Multi-head attention dynamically weights the edges during message passing. Three architectures are compared: A (fully bipartite graph), B (A with intra-set top-*k* similarity within genotype and within environment), and C (B with a single learnable supernode readout that attends over all nodes after message passing). The joint representations feed a compact MLP for yield prediction. Using a forward-time split (2014–2021 train; 2022 test with unseen genotypes and unseen environments), performance improves monotonically from A to C: A (RMSE 2.7749, PCC 0.4115, R^2^ 0.1693), B (2.3683, 0.6622, 0.4385), C (2.2120, 0.6945, 0.4823). Compared to A, C has a reduction in RMSE by 0.5629 (∼20.3%) and an increase in PCC by 0.283 (∼68.8%), indicating that global, content-adaptive aggregation promotes local G×E propagation. Performance of proposed approach remains consistent regardless of the number of genotypes per environment and has strong performance under variable or unbalanced genotype sampling expression across environments. The proposed approach is compared with methods from the Global G×E Prediction Competition and show that two of three architectures improve predictive performance, with the best architecture achieving a lower RMSE (2.2120) and a higher Pearson correlation (0.6945) than the competition-winning model.

## Author summary

This paper considers the relationship between plant genomics and environmental effects and its effect on yield. By studying a maize dataset that combines nearly 5,000 varieties of the crop in 280 location-year combinations, we make predictions on the yield of a variety when grown in a particular environment. Environmental data that is directly used in the prediction includes solar radiation, temperature, wind speed and precipitation.

To make the predictions, we use deep learning techniques that model both the temporal aspects of environmental data and the network of relationships between environmental and genomic features of the data. Our most successful model indicates that a global view of both the environmental and genomic data captures the best information for predicting yield. With this deep learning model, we can make predictions on unseen maize varieties in new environmental conditions, demonstrating the strength of our approach in modelling genotype-environment interaction.

## 1 Introduction

Global food security critically depends on functioning agricultural systems. However, it is being compromised under the pressures of increasing global population and accelerating climate change, characterized by more frequent and severe droughts and heat waves. The United Nations projects the world population to reach 9.7 billion by 2050 [1]. Additionally, 2024 was the warmest year on record [2]. Harvest data have long been the standard benchmark for evaluating crop performance. Yet, a key limitation is that year-to-year variability in environmental drivers makes such records insufficient for predicting the performance of future crops under changing conditions [3,4]. Therefore, farmers and policymakers require effective and rapid methods to assess yield at the regional scale to enable pre-allocation of inputs and informed risk management decisions.

With the advancement of next-generation sequencing technologies, dense genome-wide markers (SNPs) can now be collected cost-effectively, allowing genomic selection to estimate breeding values from genomic data only and accelerate genetic gain [5]. Through estimates of allele sharing between individuals, genomic markers allow understanding of the relationship between varieties, thus providing informative genotypic features for predicting agronomic performance [6].

The environment in which a particular variety is grown is also important. The field crop environment comprises atmospheric conditions, soil properties and management practices, including precipitation, temperature, solar radiation, wind, humidity, photoperiod, soil electrical conductivity and pH, as well as practices such as planting patterns, irrigation, fertilizer applications and pest management. Because these drivers vary between seasons and locations, models that do not account for environmental variability are likely to not accurately predict yield in all situations and perform poorly under extreme climatic conditions [7].

Yield emerges from G×E interactions, a phenomenon whereby varieties producing desired quantitative trait values in one environment may fail to provide the same outcomes in another environment [8]. It is therefore beneficial to model both genomic markers and environmental variables simultaneously when predicting yield. Consequently, any tools developed to aid crop breeding may benefit from replicating the impact of G×E by incorporating both genetic and environmental information.

Many solutions have been proposed to address crop yield prediction using G×E interactions [9–14]; however, a substantial research gap still exists in effectively capturing their interaction. Most existing methods struggle to encode environmental features for growing season and in this paper expand their approach to apply to unseen genotypes and unseen environments. Another major challenge lies in modeling large-scale datasets with thousands of genotype and environment records, robust feature representation. Brault et al. [15] showed that using genotype by environment information can improve prediction for unseen genotypes and unseen environments.

This paper propose a G×E modeling framework that encodes daily weather data using long short-term memory networks (LSTM) and genotypes using principal component analysis (PCA) into compact embeddings to predict yield of the maize. This study hypothesize that jointly learning compact representations of genotypes and environments and modeling their interactions within an attention-based graph framework can improve yield prediction performance. Subsequently using both through a graph neural network with multi-head attention to learn G×E interactions. Three graph architectures are introduced: A (fully bipartite graph), B (A with intra-set top-k similarity within genotype and within environment), and C (B with a single learnable supernode for readout). The framework is assessed under a forward-time train/test split with unseen genotypes and environments. The Genomes to Fields (G2F) G×E prediction competition dataset was used, which are publicly available [16]. Results demonstrated a consistent improvement from A to C and robustness to uneven genotype sampling per environment. Performance is compared across the three architectures and with the G2F G×E Competition results. The primary contributions of this manuscript are as follows:

- **First G**×**E graph modeling:** This work is the first GNN-based framework that models both genotype and environment information to predict crop yield.
- **Unique environmental representation:** A compact representation of season-long harvest-related weather data is presented using an LSTM, allowing the model to capture environmental context more effectively than previous approaches.
- **Progressive attention-weighted graph densification:** Modeling begins with a GNN baseline to capture G×E interactions and then systematically increase edge density to assess the impact of graph connectivity and model complexity on yield prediction accuracy.

The remainder of this paper is structured as follows. Section 2 reviews related work on G×E modeling and crop yield prediction methods. Section 3 describes the proposed framework and graph architectures. Section 4 presents the results and comparative analyses. Section 5 discusses the results and the findings. Section 6 concludes the paper and outlines future research directions.

## 2 Literature review

Several approaches have been proposed for crop yield prediction, focusing on G×E interactions, ranging from statistical models to advanced deep learning methods.

Genomic best linear unbiased prediction (GBLUP) is a linear mixed model for yield prediction in multi-environment trials [9,17,18], and can be extended to capture G×E interactions. As an example, Jarquín et al. [9] introduced a reaction norm model where genetic and environmental gradients are described as linear functions of markers and environmental covariates, respectively. Their method used covariance structures to model interactions between high-dimensional sets of markers and environmental covariates. More recently, Kunwar et al. [19] showed that using genomic prediction with environmental covariates and secondary traits can improve predictive ability, further confirming the importance of integrating environmental information into linear genomic prediction frameworks.

A limitation of linear models such as GBLUP is that they capture only linear relationships between genotype and environment, often failing to represent complex non-linear genotype × environment interactions [20]. To better capture high-order G×E interactions, researchers have therefore shifted to the use of nonlinear machine-learning methods.

Tree-based ensemble methods such as random forests and gradient boosting have been used to yield prediction problem [10,21,22]. For example Fernandes et al. [10] used LightGBM and tree-based feature partitioning to model non-linear G×E interactions.

Deep learning methodologies have been introduced that can handle the high dimensionality of input data and capture complex, nonlinear feature interactions. These models are increasingly applied in genomic selection and crop yield prediction within agricultural research. Convolutional neural networks (CNNs) are one from of deep learning used to model G×E for crop-yield prediction [11,23,24].

Feng et al. [11] presented a CNN-based based approach that processes environmental data through a CNN branch to capture temporal and spatial patterns, while genotypic marker data is fed into a separate CNN to extract genomic features. These two feature representations are then merged in a joint layer. The combined features are passed through another MLP as a predictor for yield prediction.

Recurrent neural networks, in particular, LSTM networks, have been preferred for modeling time series weather data and other sequential inputs [25]. LSTMs suitably model long-term dependencies in climate sequences in a growing season that are very important for understanding how drought periods or temperature fluctuations at different growth stages affect final yield. Zhong et al. [26] employed a spatio-temporal deep learning framework based on LSTM, integrating remote sensing and climate data to predict maize yield and detect extreme yield losses. However, this approach used only environmental features. Wang et al. [27] showed that a stacked LSTM using time-series and meteorological data improved sugar beet yield and quality prediction compared with traditional machine-learning methods.

Attention mechanisms have been also implemented on RNNs to help determine the most effective time windows or features for forecasting [28]. Shook et al. [12] proposed an LSTM–RNN with attention that combined genotype clusters and weather time-series to predict maize yield. The LSTM captured temporal patterns, while the attention layer highlighted the most important periods of the growing season, improving the model’s ability to represent G×E interactions. CNN-LSTM hybrid models have also been utilized for spatial and temporal pattern recognition [29].

Yao et al. [13] proposed GEFormer, a G×E interaction-based genomic prediction method that used both genomic and environmental data in a unified architecture. It employed a MLP to capture global and local patterns in genomic marker data. To handle temporal dependencies in weather and other environmental factors, GEFormer incorporated a linear attention mechanism, which improved the model’s ability to focus on critical time windows.

In this study, GNNs are adopted for modelling G×E. GNNs have previously been adopted for genomic selection without environmental data. He et al. [30] introduced HGATGS, a graph attention network designed for genomic selection. The method represented individuals as nodes connected by edges built from genomic similarity, enabling the model to exploit higher-order relationships among genotypes. Through graph convolution and attention mechanisms, HGATGS adaptively weights genetic contributions. Kihlman et al. [31] proposed sub-sampling graph neural networks (GCN-RS) for genomic prediction of quantitative traits. They constructed graphs that represent genomic relationships among individuals and applied graph convolutional layers to propagate marker information. To improve scalability, the sub-sampling strategy reduces graph size while retaining key genetic structure, allowing efficient training on large datasets. Both of these graph-based approaches rely only on genotype sequences and do not incorporate environmental features.

Transformers are another deep learning method used in genomic prediction and yield prediction [32,14]. As an example, Zou et al. [14] assembled a large-scale multi-environment dataset wheat yield records, paired with real-valued environmental data. They propose a multi model deep learning framework that uses both Bi-LSTM and Transformer architectures to model temporal environmental variables. Genotype data are encoded in a parallel branch, and the learned genotype and environment embeddings are fused for trait prediction.

Despite significant progress, existing G×E modeling approaches still have important limitations. Linear models are restricted to simple relationships, while many machine learning and deep learning methods combine genotype and environment information without directly modeling their interactions. Graph neural networks have previously been applied mainly to genotype data and typically do not incorporate environmental information. These limitations motivate the need for a unified framework that use both genotypes and environments and captures their interactions in a structured manner.

## 3 Materials and methods

This section describes the theoretical foundations, dataset, preprocessing procedures, and model architectures used in this paper.

### 3.1 Graph foundations

Graphs are used to model complex and interconnected systems in the real world, such as knowledge graphs, social networks, and molecular structures. Formally, a graph *G*=(*V*,*E*) is a set *V* of *n* nodes connected by edges. *A* denotes an *n* × *n* adjacency matrix where each entry $a_{ij}\in \{0,1\}$ indicates the presence or absence of an edge connecting nodes *i* and *j*. Additionally, ${𝒩}_{i}$ denotes the set of neighbors of node *i*, which are the nodes connected to *i* by an edge, i.e., ${𝒩}_{i}=\{\;j\in V|a_{ij}=1\;\}$.

### 3.2 Message-passing graph neural networks

GNNs are a class of deep learning architectures designed to operate on graph-structured data. GNNs leverage the graph topology to propagate and aggregate information between connected nodes. Formally, the node embedding $h_{i}^{\left(\ell \right)}$ of each node i∈V is updated from layer ℓ to ℓ+1 via a two-step message-passing process:

(1) Message construction:
  For each node *i* and its neighbors $j\in {𝒩}_{i}$, a message $m_{ij}^{\left(\ell \right)}$ is constructed to capture the relationship between the embeddings of nodes *i* and *j*:
  $$\begin{array}{c} m_{ij}^{\left(\ell \right)}=\psi \;(h_{i}^{\left(\ell \right)},\;h_{j}^{\left(\ell \right)}),\;j\in {𝒩}_{i}, \end{array}$$ (1)
  where ψ(·) is a problem-dependent function used to construct edge messages.
(2) Neighbor aggregation:
  All messages from the neighbors of node *i* are then combined to produce a single aggregated message $m_{i}^{\left(\ell \right)}$:
  $$\begin{array}{c} m_{i}^{\left(\ell \right)}=\underset{j\in {𝒩}_{i}}{⨁}m_{ij}^{\left(\ell \right)}, \end{array}$$ (2)
  where ⨁ denotes an aggregation operator, such as sum, mean, or max, applied over all neighbors $j\in {𝒩}_{i}$, and the node embedding is then updated as
  $$\begin{array}{c} h_{i}^{\left(\ell +1\right)}=m_{i}^{\left(\ell \right)}. \end{array}$$ (3)
• this message passing allows each node to incorporate information from its neighbors, so genotype and environment embeddings gradually become interaction-aware across layers during iterations [33,34].

### 3.3 Prediction from embeddings

Initially every node is assigned its raw embedding. Equations (1)–(3) iteratively refine these embeddings, $h_{i}^{\left(0\right)}\;\to \;h_{i}^{\left(1\right)}\;\to \;\ldots \;\to h_{i}^{\left(L\right)},$ incorporating increasingly larger neighborhood context. The final embedding matrix $H^{\left(L\right)}=[h_{1}^{\left(L\right)}\;\ldots h_{n}^{\left(L\right)}{]}^{\;⊤}$ serves as input predictor:


$$\begin{array}{c} \hat{y}=g\;(\;H^{\left(L\right)}), \end{array}$$ (4)

where *g* denotes a predictor such as a linear classifier, bayesian regression head or multilayer perceptron.

### 3.4 Graph attention network

A class of GNNs uses attention mechanisms to weight the importance of different neighbors during aggregation [35]. Graph attention network refine (1)–(2) by assigning *learned, data dependent* weights to each neighbor:


$$\begin{array}{c} \alpha_{ij}^{\left(\ell \right)}=\frac{\exp\;((W_{Q}^{\left(\ell \right)}h_{i}^{\left(\ell \right)}{)}^{\;⊤}W_{K}^{\left(\ell \right)}h_{j}^{\left(\ell \right)})}{\sum_{j^{\prime }\in {𝒩}_{i}}\exp\;((W_{Q}^{\left(\ell \right)}h_{i}^{\left(\ell \right)}{)}^{\;⊤}W_{K}^{\left(\ell \right)}h_{j^{\prime }}^{\left(\ell \right)})}, \end{array}$$ (5)

$$\begin{array}{c} m_{ij}^{\left(\ell \right)}=\alpha_{ij}^{\left(\ell \right)}\;W_{V}^{\left(\ell \right)}h_{j}^{\left(\ell \right)}, \end{array}$$ (6)

where $W_{Q}^{\left(\ell \right)},W_{K}^{\left(\ell \right)},W_{V}^{\left(\ell \right)}\in {\mathbb{R}}^{d\times d}$ are learned projections. The node update thus becomes


$$\begin{array}{c} h_{i}^{\left(\ell +1\right)}=h_{i}^{\left(\ell \right)}+\sum_{j\in {𝒩}_{i}}m_{ij}^{\left(\ell \right)}. \end{array}$$ (7)

Graph Attention Networks (GATs) are employed as main model to represent G×E in Section 3.8. The attention mechanism allow the model to learn which neighboring nodes are more important, which makes interaction strength to vary across different genomic–environmental node pairs.

### 3.5 Dataset

**Fig 1:**
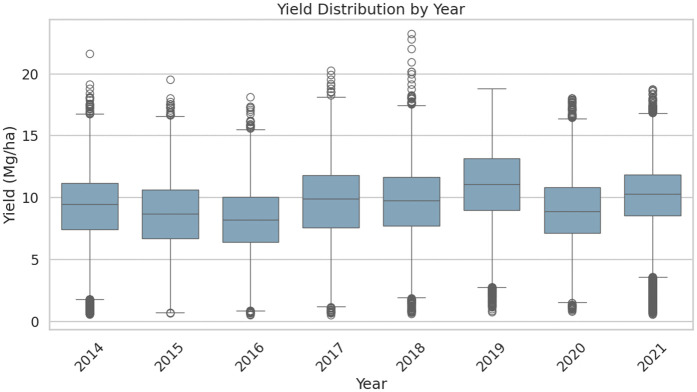
Distribution of maize yield (Mg/ha) across training years (2014–2021).

The Genomes to Fields (G2F) Genotype × Environment Prediction Competition data were utilized [16], which is a large multi-year maize field trial resource and is specifically designed to benchmark G×E models. In this paper, the environment corresponds to a site-year combination and a hybrid denotes a maize genotype. The 2023 release has over 180,000 individual field plots(experimental unit in which a specific hybrid is grown within a given environment) which are assessed under nearly 280 unique year-location combinations in North America. In total, the experiment comprised 4,683 distinct hybrids in the training dataset, while 4,928 hybrids are present in the full genotypic dataset. The overall mean yield is 9.52 Mg/ha with a standard deviation of 2.98 Mg/ha. The distribution of yield across years is shown in Fig 1. For 35,901 hybrid–environment combinations with repeated yield measurements, the same hybrid was harvested multiple times in the same environment. The median within-pair standard deviation among those yield values was 0.93 Mg/ha, shows inherent variability even under same genotype and environmental conditions.

#### 3.5.1 Phenotypic data.

Every field plot has central agronomic characteristics such as grain yield (converted to 15.5% moisture, given in Mg ha^−1^), days to anthesis, days to silking, and the anthesis-silking interval (ASI). Anthesis refers to the stage when maize plants release pollen from the tassel, while silking marks the emergence of silks from the ear that receive pollen. The anthesis–silking interval represents the time difference between these stages and is associated with pollination success and yield performance [36]. The trials were performed according to standard management practices, and the majority of hybrids were replicated in several plots in the environments. The summary statistics indicate that considerable variability exists not only between environments (like the differences in the average yield from one site to another) but also within environments (the variation among replicates), making it a realistic challenge for modelling.

#### 3.5.2 Genotypic data.

Through the use of publicly available parental marker profiles, hybrid genotypes were deduced. The final marker matrix consists of 437,214 high-quality SNPs coded as 0, 1, or -1 that were recorded in the 4,928 distinct hybrids. The filtering process eliminated SNPs with a minor allele frequency below 0.01 or a missingness above 5%.

#### 3.5.3 Weather data.

The weather data were obtained from the G2F competition dataset [16], which provides daily meteorological records for each site-year pair. These records were originally retrieved from the NASA POWER archive and include radiation variables (All Sky Surface PAR Total, All Sky Surface Shortwave Downward Irradiance, and All Sky Surface Shortwave Downward Direct Normal Irradiance), temperature measures (wet bulb, maximum, minimum, and mean at 2 m), humidity indicators (specific and relative at 2 m), dew/frost point (2 m), precipitation corrected, surface pressure, wind speed (2 m), and soil moisture metrics at multiple depths (profile soil moisture, surface soil wetness, and root zone soil wetness). All features were standardized to zero mean and unit variance within each environment before modeling to ensure comparability across sites and seasons.

#### 3.5.4 Cross-validation and data splitting.

In order to imitate actual breeding, a forward-time validation strategy was employed, in which all models were trained on field-trial data from 2014 to 2021. The 2022 trials, on the other hand, were reserved as an independent test set and were not used at any stage of training or validation; they contain unseen genotypes and unseen environments. During the 2014–2021 period, an 80/20 split was used to create training and validation subsets. It was ensured that no identical genotype–environment combination appeared in both subsets, thus providing an estimate of performance on unseen contexts during model development. The 2022 trials, which were not used at any point during model development, were used only for the final evaluation, showing the ability of each model to apply the learned G×E relationships to a new season with new genotypes.

### 3.6 Genotype dimensionality reduction

In order to cope with the high dimensionality of genotype dataset (4,928 samples by 437,214 SNPs), A two-step reduction strategy was applied. First, probabilistic imputation was performed to replace missing entries: for each SNP column, The empirical frequencies were estimated of {−1,0,+1} and stochastically sampled replacements according to these probabilities. This approach preserves each marker’s marginal distribution without imposing strong assumptions on linkage structure. Subsequently, classic variance-based and redundancy filters were used to remove uninformative or SNPs with very high correlation:

(1) Low-Variance Filter: SNP columns were removed if that had a single genotype value in at least 95% of the observations.
(2) Windowed Similarity Pruning: To reduce local redundancy, the SNP sequence was processed using sliding windows of size *L*, where *L* represents the number of SNP markers per window. Within each window, similarity was calculated between SNP columns, and when two SNPs were identical in at least 95% of samples, only one representative marker was retained. Thus, local redundancy was reduced while preserving regional structure.

These filters reduced the feature set from 437,214–133,673 SNPs. The second stage consisted of performing PCA on this filtered matrix. PCA is the identification of orthogonal axes with the most variance which automatically compresses correlated markers to a small number of components. The 548 retained principal components, resulted in almost 90% of the total genetic variance being accounted for. The cumulative explained variance of the genotype PCA is provided in Fig B in S1 Appendix. The resulting 548-dimensional vector provides a compact yet informative summary of each genotype, lowering computational demands and mitigating overfitting risks associated with excessively long input sequences.

### 3.7 Weather-to-harvest feature extraction using LSTM

Among the weather variables mentioned in section 3.5.3 five variables were kept: All–Sky Surface PAR Total, 2 m Maximum Temperature, 2 m Minimum Temperature, Corrected Precipitation, and All–Sky Surface Short-Wave Direct-Normal Irradiance. This small subset was chosen to span the range of environmental, representing a broad range of input types. The subset was chosen to improve computational efficiency. The choice of subset is validated by explainability methods as shown in Fig A and Table A in S1 Appendix. Accordingly, for each environment, the daily values of the five selected weather variables were organized into a k×5 matrix, where k denotes the number of growing-season days. As the environmental dataset is large, an effective summarization method is required. Previous approaches have relied on averaging (weekly or monthly) techniques [37]; however, these often conceal short but high-impact events, such as sudden heatwaves during tasseling, the stage when the maize tassel emerges and pollen is shed. To address this, Instead, the sequence of daily observations using a single-layer unidirectional LSTM is encoded.

After z-score normalising every feature, the daily vector $x_{t}\in {\mathbb{R}}^{5}$ is fed to a single-layer unidirectional LSTM using the standard formulation, whose gating equations are


$$\begin{array}{c} {𝐢}_{t}=\sigma \;\left({𝐖}_{i}{𝐱}_{t}+{𝐔}_{i}{𝐬}_{t-1}+{𝐛}_{i}\right), \end{array}$$ (8)

$$\begin{array}{c} {𝐟}_{t}=\sigma \;\left({𝐖}_{f}{𝐱}_{t}+{𝐔}_{f}{𝐬}_{t-1}+{𝐛}_{f}\right), \end{array}$$ (9)

$$\begin{array}{c} {𝐨}_{t}=\sigma \;\left({𝐖}_{o}{𝐱}_{t}+{𝐔}_{o}{𝐬}_{t-1}+{𝐛}_{o}\right), \end{array}$$ (10)

$$\begin{array}{c} {\tilde{𝐜}}_{t}=\tanh\;\left({𝐖}_{c}{𝐱}_{t}+{𝐔}_{c}{𝐬}_{t-1}+{𝐛}_{c}\right), \end{array}$$ (11)

$$\begin{array}{c} {𝐜}_{t}={𝐟}_{t}⊙{𝐜}_{t-1}\;+\;{𝐢}_{t}⊙{\tilde{𝐜}}_{t}, \end{array}$$ (12)

$$\begin{array}{c} {𝐬}_{t}={𝐨}_{t}⊙\tanh\;\left({𝐜}_{t}\right), \end{array}$$ (13)

where σ(·) is the logistic sigmoid and ⊙ denotes element-wise multiplication [38,39]. Here, $W_{i},W_{f},W_{o},W_{c}\in {\mathbb{R}}^{m\times 5}$ are the input weight matrices, $U_{i},U_{f},U_{o},U_{c}\in {\mathbb{R}}^{m\times m}$ are the recurrent weight matrices, and $b_{i},b_{f},b_{o},b_{c}\in {\mathbb{R}}^{m}$ are bias terms, where *m* denotes environmental embedding vector length. All weight matrices and biases are learnable parameters optimized during training. At the beginning of each sequence, both the hidden state **s**_0_ and the cell state **c**_0_ are initialized to zero vectors of dimension *m*, and are then updated recursively according to Equations (8)–(13). The final hidden state ${𝐬}_{k}\;\in \;{\mathbb{R}}^{m}$, which encodes the entire sequence of *k* daily records, is used as the environmental embedding **z**_env_. These equations control how information flows through time, allow the model to summarize the daily weather sequence into a fixed-length seasonal representation.

### 3.8 End-to-end G × E pipeline

**Fig 2:**
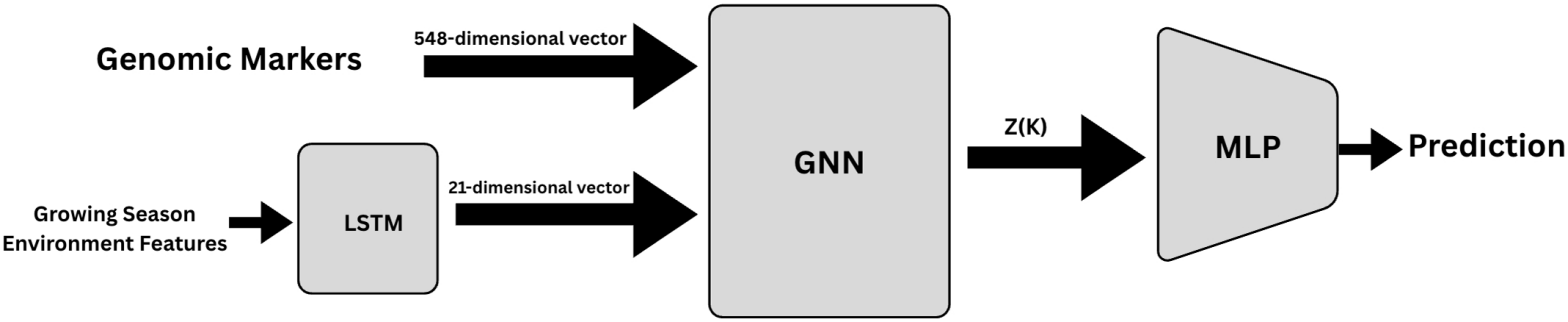
Overview of the proposed G × E prediction pipeline. Genomic markers are reduced to 548 principal components and used as genotype node features. Daily weather variables are encoded via LSTM into a 21-dimensional environment embedding. These embeddings form nodes in a GNN, followed by an MLP predictor for yield estimation.

Fig 2 presents the full G×E pipeline used for crop yield prediction. This figure brings together the individual components introduced in the sections 3.6 and 3.7 into a unified framework. Two complementary embeddings are first derived:

- **Genomic stream**: After completing the quality control and imputation processes, 437,214 SNPs are embedded to 548 principal components. The resulting output is a dense marker vector which encodes the major axes of the genetic variation.
- **Weather data set**: The single-layer LSTM reads five variables on a daily basis, i.e., total PAR, maximum temperature, minimum temperature, corrected precipitation, and short-wave DNI. The last hidden state ${𝐬}_{k}\;\in \;{\mathbb{R}}^{21}$, representing the 21-dimensional order-aware weather fingerprint, is denoted as **z**_env_.

Both embeddings are given as node features to a GNN with 21 + 548 = 569 nodes. After *K* propagation steps, updated node embeddings are obtained. The set of embeddings **Z**(*K*) are passed to the multilayer perceptron (MLP) model. **Z**(*K*) in Architectures A and B, consists of the genotype related nodes embeddings, while in Architecture C it corresponds to the supernode embedding. Further details of these architectures are provided in Sections 3.8.1–3.8.3.

The MLP maps the input embeddings to a single scalar value representing the predicted yield. The training process is based on minimizing a weighted combination of mean-squared error (MSE) and mean absolute error (MAE), with early stopping, while hyper-parameters are selected using the forward-time cross-validation procedure (2014–2021 training seasons, 2022 reserved).

Next, three variations of the GNN architecture are presented, all of which have the same interconnecting pipeline components as the basic model.

#### 3.8.1 Architecture A.

**Fig 3:**
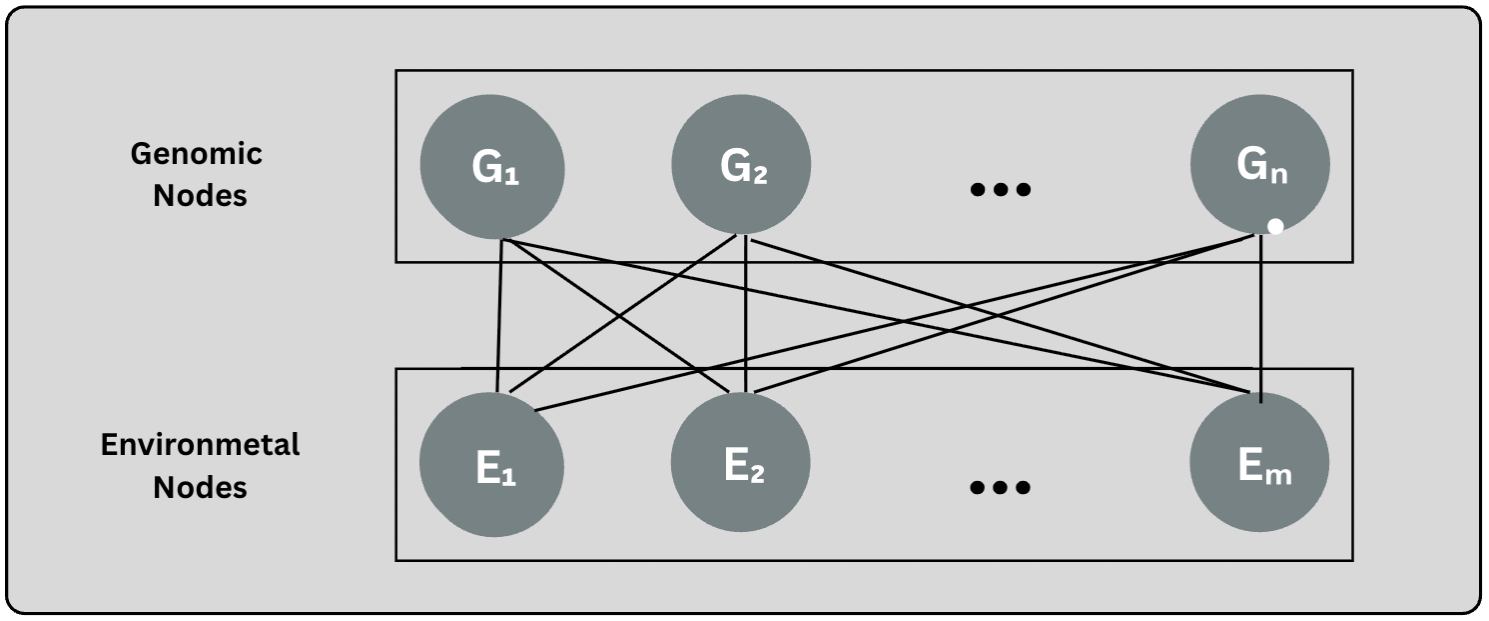
Architecture A fully connected bipartite graph between 548 genotype nodes (principal components) and 21 environmental nodes (LSTM-derived features). All genotype–environment pairs are connected via multi-head attention message passing.

Architecture A represents the interactions between genotype and environment using a fully connected bipartite graph (Fig 3), which is comprised of two distinct node sets.

- **Genomic nodes**: The *n* = 548 principal components scores from the SNP matrix are instantiated as individual nodes *G*_*i*_.
- **Environmental nodes**: The LSTM’s *m* = 21-dimensional weather fingerprint is decomposed into *m* single-feature nodes *E*_*j*_.

Each genomic node connects with each environmental node, which results in *n* × *m* edges. Communication by means of messages across each edge is done through the use of multi-head transformer attention:


$$\begin{array}{c} \alpha_{ij}^{\left(l\right)}=\frac{\exp\;(\frac{\left({𝐖}_{Q}^{\left(l\right)}{𝐫}_{i}{)}^{\;⊤}({𝐖}_{K}^{\left(l\right)}{𝐫}_{j}\right)}{\sqrt{d_{k}}})}{\sum_{j^{\prime }\in {𝒩}_{i}}\exp\;(\frac{\left({𝐖}_{Q}^{\left(l\right)}{𝐫}_{i}{)}^{\;⊤}({𝐖}_{K}^{\left(l\right)}{𝐫}_{j^{\prime }}\right)}{\sqrt{d_{k}}})}, \end{array}$$ (14)

$$\begin{array}{c} {𝐦}_{ij}^{\left(l\right)}=\alpha_{ij}^{\left(l\right)}\;{𝐖}_{V}^{\left(l\right)}{𝐫}_{j}. \end{array}$$ (15)

where **r**_*i*_ and **r**_*j*_ are the current embeddings of nodes *G*_*i*_ and *E*_*j*_, and ${𝐖}_{Q}^{\left(l\right)}$, ${𝐖}_{K}^{\left(l\right)}$, ${𝐖}_{V}^{\left(l\right)}$ are head-specific projection matrices. Messages from all heads are concatenated and aggregated at the target node; residual connections and layer normalization follow each attention block to stabilize training.

When *K* such propagation layers are stacked together it leads to genomic embeddings which incorporate the total range of genotype–environment interactions evident through the bipartite attention mechanism. Architecture A represents baseline maximal-capacity design; the following variations will either prune or re-weight the edges and accordingly, explore accuracy–efficiency trade-offs without changing any other pipeline components.

#### 3.8.2 Architecture B.

**Fig 4:**
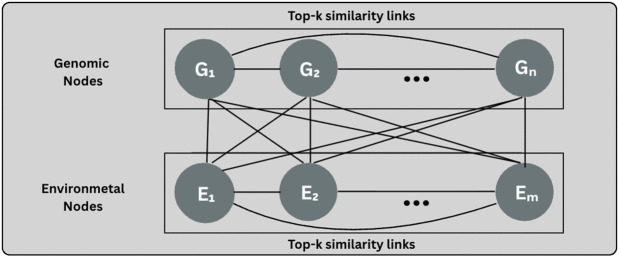
Architecture B extension of Architecture A with additional directed intra-set top-k similarity edges (k = 10) among genotype nodes and among environment nodes, while retaining all bipartite genotype–environment connections.

The extension of the bipartite attention framework with Architecture B is done by including intra-set connectivity on both the genotype and environment sides while still keeping all *n* × *m* genotype–environment edges (Fig 4). This improves the information exchange between the two modalities and within each modality, allowing locally conducive embeddings to exchange information during cross-set propagation.

The *n* = 548 PCA components that are extracted from the SNP matrix represent the genomic nodes *G*_*i*_. Each *G*_*i*_ remains connected to the environmental nodes, and in addition to this, each node *G*_*i*_ is attached to its top-*k* most nearest genomic neighbors (*k* = 10) according to


$$\begin{array}{c} s(u,v)=\frac{1}{1+|x_{u}-x_{v}|} \end{array}$$ (14)

where *u* and *v* refer to individual nodes, and *x*_*u*_ and *x*_*v*_ denote their associated embedding vectors.

The *m* = 21 single-feature weather nodes *E*_*j*_ also conserve their full bipartite links to genomic nodes while each *E*_*j*_ forms the top-*k* most similar environment neighbors (*k* = 10) using equation 14, which thus generates a intra-environment adjacency with per-node out-degree *k*. These intra-set edges are not intended to represent redundancy among nodes. Instead, they provide a local graph structure that supports information propagation among nodes with similar representations during message passing.

Let *A*_*GG*_ and *A*_*EE*_ symbolize the directed intra-set adjacencies (bounded out-degree *k* per node), and *A*_*GE*_, *A*_*EG*_ the bipartite adjacencies between *G* and *E*. The complete message-passing graph is symbolized as


$$\begin{array}{c} {}[\begin{array}{cc} A_{GG} & A_{GE}\\ A_{EG} & A_{EE} \end{array}], \end{array}$$

where all of the *n* × *m* bipartite edges of architecture A is present, and the intra-set edges are additionally included.

Message passing is accomplished with the aid of multi-head attention operating over all incident edges. Attention coefficients are computed on any edge (be it intra-set or cross-set), and messages are aggregated through residual connections and layer normalization after each block (as in architecture A). The extra connectivity allows for mixed multi-hop paths; for example, $G_{1}\to G_{2}$ (intra-genotype) and $G_{2}\to E_{1}$ (bipartite) mean that at the end of two propagations, *E*_1_ has obtained information from *G*_1_. More generally, after ℓ propagations, a node’s embedding brings together all members of its ℓ-hop neighborhood according to the block adjacency, using both intra-set smoothing and inter-set interaction.

This approach captures a similarity-driven local structure while also keeping the full genotype–environment map from the architecture A.

#### 3.8.3 Architecture C.

**Fig 5:**
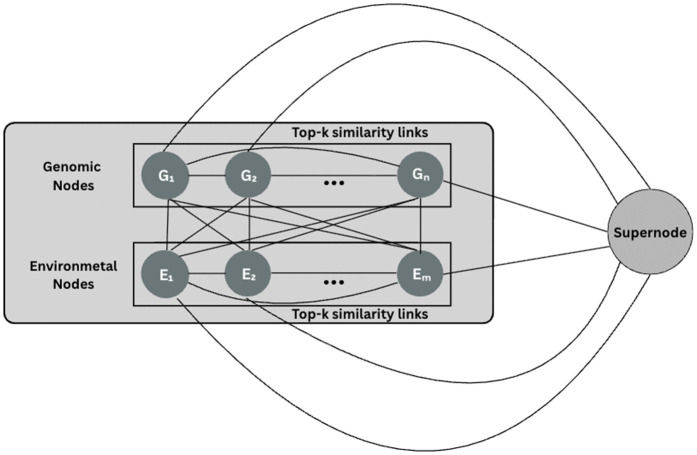
Architecture C retains the message-passing structure of Architecture B and introduces a global supernode attention readout applied after K propagation layers. The supernode attends to all genotype and environment embeddings to produce a compact graph-level representation used for prediction.

Architecture C changes the graph-level readout, which is the same as the single block-graph, node-level G×E message passing of Architecture B (intra-genotype and intra-environment edges plus full bipartite G↔E). This architecture replace the forwarding of all genomic node embeddings to the MLP with a single learnable supernode query that attends to all nodes and aggregates them into one global embedding via multi-head attention (Fig 5).

Let $G=\{G_{i}{\}}_{i=1}^{n}$ and $E=\{E_{j}{\}}_{j=1}^{m}$ represent genotype and environment nodes, respectively. To create devoted directed top-*k* intra-set adjacencies *A*_*GG*_ and *A*_*EE*_ we use equation 14, and keep all bipartite edges *A*_*GE*_ and *A*_*EG*_.

After *K* iterations of message passing, the model obtains updated embeddings for the *n* genotype nodes and *m* environment nodes, denoted by ${𝐇}_{g}^{\left(K\right)}$ and ${𝐇}_{e}^{\left(K\right)}$. With the help of multi-head attention, the supernode query *s* conveys content-dependent weights to each node, allowing the model to highlight the most informative genotype and environment signals. Formally, the attention weights are defined as:


$$\begin{array}{c} \alpha_{g}=\mathrm{softmax}\;\left(\frac{(𝐬{𝐖}_{Q})({𝐇}_{g}^{\left(K\right)}{𝐖}_{K}{)}^{⊤}}{\sqrt{d_{k}}}\right),\;\alpha_{e}=\mathrm{softmax}\;\left(\frac{(𝐬{𝐖}_{Q})({𝐇}_{e}^{\left(K\right)}{𝐖}_{K}{)}^{⊤}}{\sqrt{d_{k}}}\right), \end{array}$$ (17)

and the weighted sums yield


$$\begin{array}{c} {𝐳}_{g}=\alpha_{g}\;{𝐇}_{g}^{\left(K\right)}{𝐖}_{V},\;{𝐳}_{e}=\alpha_{e}\;{𝐇}_{e}^{\left(K\right)}{𝐖}_{V}, \end{array}$$ (18)

where ${𝐖}_{Q},{𝐖}_{K},{𝐖}_{V}$ are projection matrices and *d*_*k*_ is the key/query dimension. Finally, the two summaries are concatenated to form


$$\begin{array}{c} 𝐳=[{𝐳}_{g};{𝐳}_{e}], \end{array}$$ (19)

which serves as a compact representation of the genotype–environment graph and is passed to the MLP predictor. The above attention formulation computes normalized compatibility scores between the supernode and each nodes, and uses them to create embedding for prediction.

The supernode readout is global and can attend to all nodes while remaining content-adaptive, as the weights depend on the learned attention mechanism. The supernode attention is executed after all message-passing layers have finished; that is, it operates on the final node embeddings that already incorporate genotype–environment context. This ensures that the global summary captures both local neighborhood effects and higher-order multi-hop relationships that were propagated through the GNN.

### 3.9 Hyper parameter tuning

All architectures were tuned under the same protocol described in Secs. 3.8.1–3.8.3: forward-time validation (2014–2021 train, 2022 test). The hyper parameters are described in Table 1. To ensure a controlled comparison across architectures, most shared hyperparameters were kept identical unless required by the graph design. AdamW was used as the optimizer, which is a variant of Adam that decouples weight decay from the gradient-based parameter updates, often improving regularization stability during training. The loss function was defined as λMSE+(1−λ)MAE, where λ=0.8.

**Table 1:** Final training configuration across architectures. A uses only bipartite edges (no intra-set *k*); B adds intra-set edges; C keeps B’s message passing but replaces readout with a single global supernode attention pooling computed *after* message passing.

Hyperparameter	Arch A	Arch B	Arch C
Batch size	32	32	32
Epochs	100	100	100
Message-passing rounds *K*	30	30	30
Intra-set *k* (*A*_*GG*_, *A*_*EE*_)	— (not used)	10	10
Hidden dim *d*	128	128	128
Attention heads	8	8	8
Learning rate	3 × 10^−4^	3 × 10^−4^	3 × 10^−4^
Weight decay	1 × 10^−4^	1 × 10^−4^	1 × 10^−4^
Workers	4	4	4
MLP (3-layer architecture)	256–128–64–1	256–128–64–1	256–128–64–1
Optimizer	AdamW	AdamW	AdamW
Loss	λMSE+(1−λ)MAE	λMSE+(1−λ)MAE	λMSE+(1−λ)MAE

## 4 Results

This section provides the experimental evaluation of the proposed architectures, comparing predictive performance across models and analyzing performance under unseen genotypes and environments.

### 4.1 Environmental embedding dimension selection

**Fig 6:**
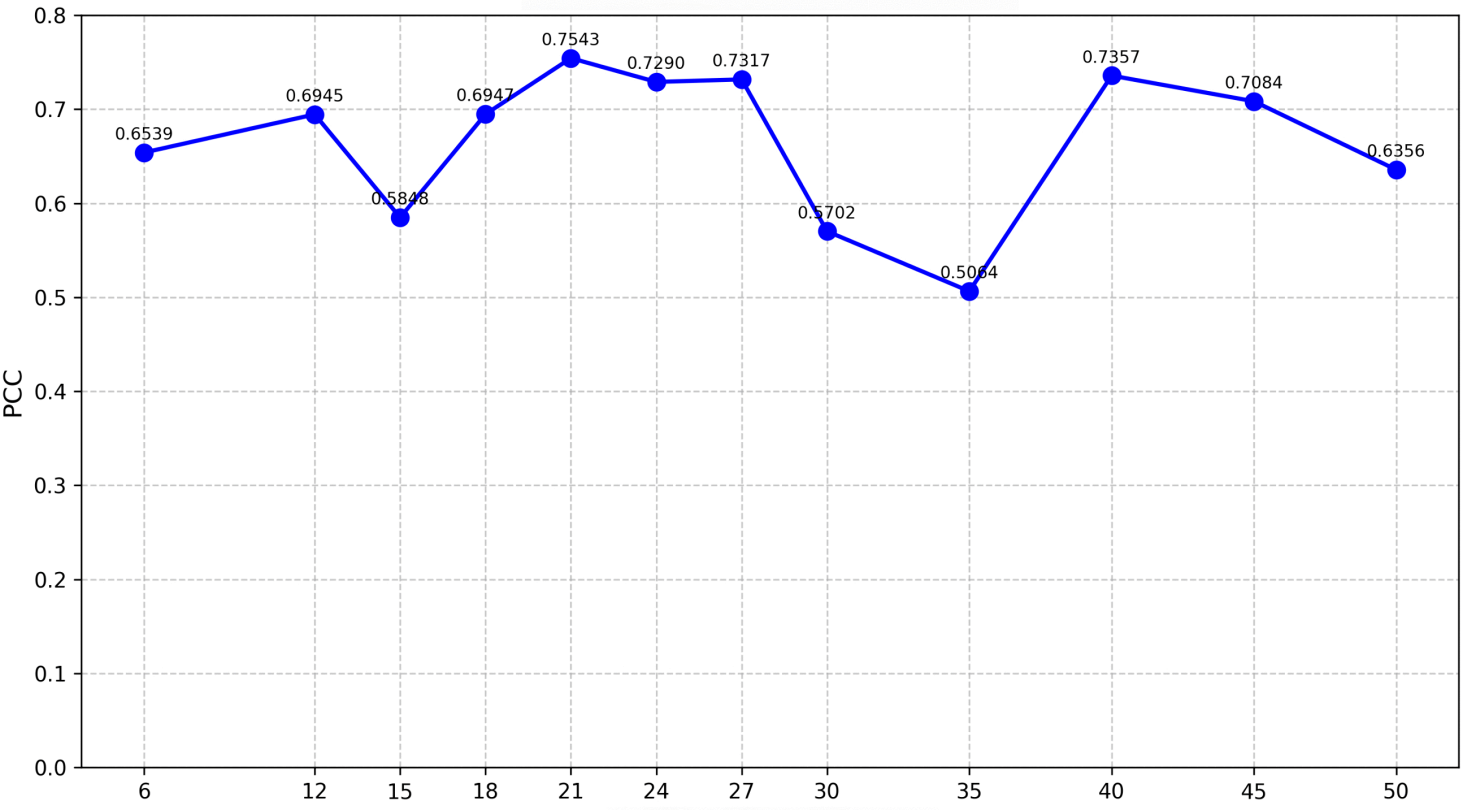
Pearson correlation coefficient (PCC) on the validation set as a function of environmental embedding dimension m using Architecture A. Each point represents average PCC across genotype–environment pairs. Performance peaks at *m* = 21.

To choose the embedding length values of m∈{6,9,12,15,18,21,24,27,30,35,40,45,50} were considered. For each embedding size *m*, the environmental representation **z**_env_ was used to predict yields across all genotype–environment pairs in the validation set, and performance was evaluated using the Pearson correlation coefficient (PCC) between predicted and observed yields. Fig 6 shows that performance peaks at *m* = 21 (PCC ≈ 0.75) using architecture A, plateaus for *m* = 24–27, and then generally declines once *m* ≥ 30. Therefore, *m* = 21 was fixed for all downstream genotype environment fusion models [28], giving us a compact but information-rich representation that captures normal behavior of weather data during the growing season [40].

### 4.2 Architectural comparison

The performance of three GNN designs was evaluated that were trained under the same data splits and training conditions: A (fully bipartite), B (bipartite + intra-set edges), and C (the same message passing as in B but with a global supernode readout applied after the message passing). The performance was evaluated using RMSE, PCC, and *R*^2^ over the unseen gentypes and unseen environments as a test-set.

**Fig 7:**
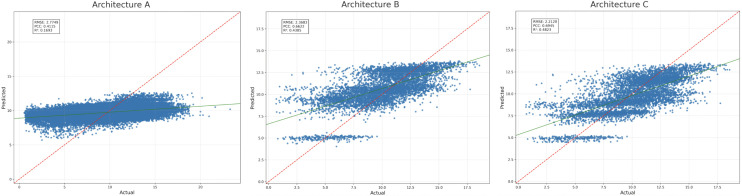
Predicted vs. actual yield on the test set for Architectures A, B, and C (left to right). The red dashed line is the identity; the green line is the fitted regression. Each point in the plot refers to an individual genotype-site-year combination in the test set.

For the three architectures A, B and C under consideration, the increasing tendency can be seen in the performance with regard to accuracy and correlation (Table 2 and Fig 7). A, posts RMSE = 2.7749 and PCC = 0.4115. B, the error reduces to RMSE = 2.3683 and the PCC is boosted to 0.6622 which is 14.7% less error and 0.25 higher PCC than A. C, attains RMSE = 2.2120 and PCC = 0.6945 which is 20.3% less error than A and 6.6% less than B, with the highest correlation overall. The scatter plots for A, B and C show the strongest relationship between actual and predicted yield for C.

**Table 2:** Test-set metrics across architectures.

Metric	A	B	C
RMSE ↓	2.7749	2.3683	**2.2120**
PCC ↑	0.4115	0.6622	**0.6945**
*R*^2^ ↑	0.1693	0.4385	**0.4823**

**Fig 8:**
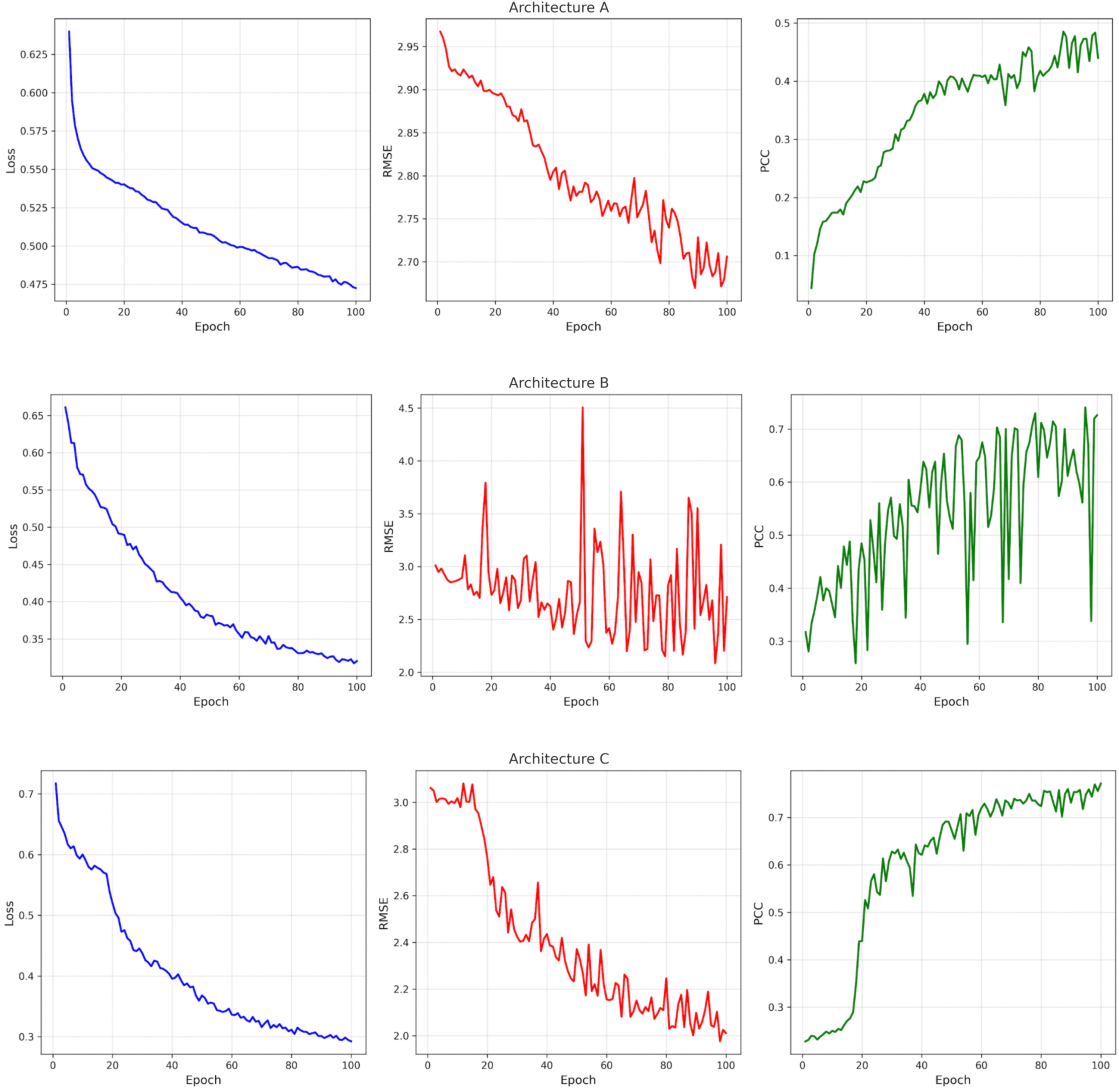
Training dynamics for A (top), B (middle), and C (bottom). Each row shows training loss (left), RMSE (middle), and PCC (right) across epochs.

The steady progress of all three models in the training curves shown in Fig 8 is very evident. A shows a gradual improvement in both RMSE and PCC as the epochs progress. B obtains quicker results in PCC, however it has to sacrifice a little stability in RMSE during training. C has the clearest stage: an improvement phase during epochs ∼15–25 followed by a stable plateau at lower RMSE and higher PCC.

### 4.3 Site - year analysis

**Fig 9:**
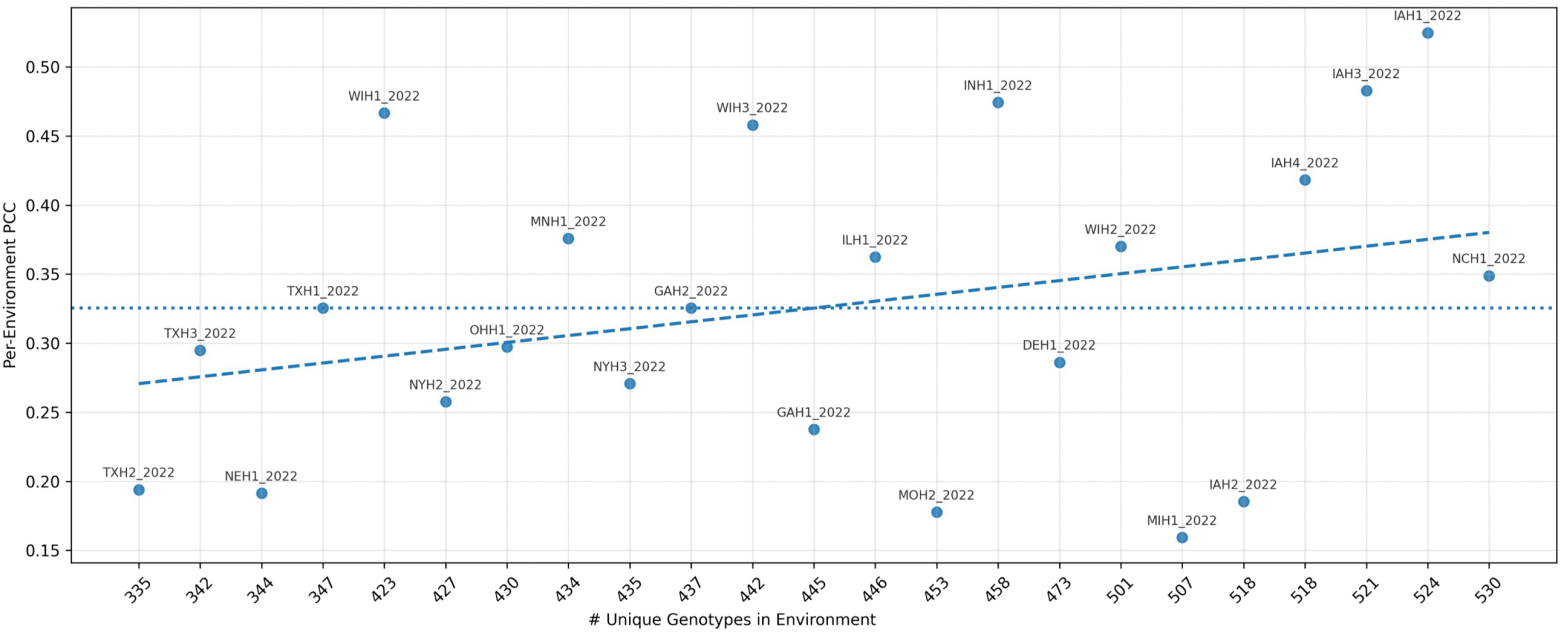
Per-environment predictive correlation (PCC) versus the number of unique genotypes evaluated.

Architecture C was used, and performed the evaluation across all environments in the test set, which included genotype counts ranging from 335 to 530 (Fig 9). Although some environments show variability in PCC, the fitted regression line indicating no strong association between sample size in the environment and predictive accuracy. Thus, the model’s environment-level predictive ability appears independent of genotype count; increasing the number of genotypes in an environment (without changing covariates) does not automatically yield a higher PCC. Moreover, environments with larger numbers of genotypes are not influencing the system at the expense of those with fewer genotypes.

**Fig 10:**
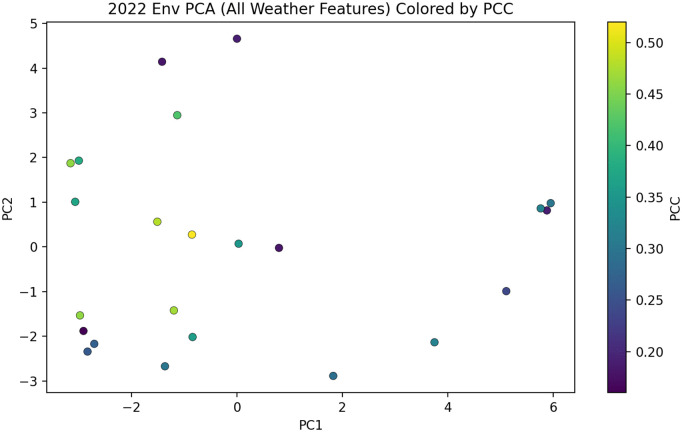
PCA of weather features for test environments. Each point represents one environment projected onto the first two principal components (PC1 and PC2). Colors indicate the mean PCC achieved by the proposed model in that environment.

All 2022 test environments were projected into a two-dimensional PCA space derived from aggregated weather features and colored each environment according to its mean PCC. As shown in Fig 10, most environments are distributed near the center. No clear performance differentiation is observed for environments located in more extreme positions. This suggests that the model primarily captures average environmental patterns, while its ability to explicitly account for extreme climatic conditions remains limited.

### 4.4 Benchmarking against the G2F G×E Competition

The proposed method was evaluated on the identical Genomes to Fields (G2F) dataset [16] and split utilized in the public G×E yield-prediction competition (2014–2021 training; 2022 held-out test). The competitive leader-board is based on the average RMSE values and Pearson correlation (PCC) of different environments. As a transparent external baseline, Table 3 shows the three proposed models (A–C) with the top competition results.

**Table 3:** Comparison of proposed architectures with top models from the Global G×E Prediction Competition using the same forward-time split (2014–2021 train, 2022 test).

Model	RMSE ↓	PCC ↑
CLAC (winner)	2.458	0.631
DataJanitors (best PCC)	2.752	0.644
Architecture A	2.7749	0.4115
Architecture B	2.3683	0.6622
Architecture C (proposed, best)	**2.2120**	**0.6945**

The best performing model, Model C, has achieved RMSE = 2.2120 and PCC = 0.6945, thus, clearly surpassing the winner of the competition (CLAC) who obtained across-environment RMSE = 2.458; PCC = 0.631 and was the best reported across-environment PCC (DataJanitors; PCC = 0.644).

## 5 Discussion

These findings indicate that graph neural networks are capable of capturing G×E interactions. By representing genotypes and environments as nodes within a graph, the GNN can model the complex dependencies between them. Consequently, the GNN was able to identify the G×E patterns, leading to improved yield predictions in performed experiments.

The combination of local and global graph edges was one of the key factors that contributed to the success of the model in modeling G×E interactions. Intra-set edges were incorporated among genomic and environmental nodes, which allows the model to exchange information between connected nodes and exploit similarities among related nodes within both domains.

A supernode was added that connects to every other node in the graph, helping the model learn global relationships. Through this design, the model captures higher-order similarities that exist among all genotypes or environments. Together, the local and global graph structures allow the model to better capture G×E interactions.

An LSTM was used to encode environmental time series data. Instead of a standard description of environmental features based on static summary statistics, sequential daily weather data were provided to the LSTM (temperatures, precipitation, etc.) to an LSTM resulting in the condensed version of the growing season. The primary idea is that timing and duration of environmental events may influence crop yield in ways that cannot be explained by average conditions alone.

Incorporating local subgraphs among genomic and environmental nodes increases the model’s complexity and allows better information exchange. These subgraphs help capture interactions within the genomic and environmental nodes, allowing them to share information within their groups and help the entire of the architecture to better model G×E interactions.

The proposed framework shows several strengths. The integration of LSTM-based temporal encoding with attention-guided graph modeling allows structured learning of G×E interactions under unseen genotypes and environments. Despite the good predictive performance, several limitations should be acknowledged. As shown in Fig 8, most test environments cluster near the center of the PCA space, and no clear performance differentiation is observed for environments located in more extreme climatic regions, suggesting limited sensitivity to extreme conditions. In addition, this study primarily focused on environmental representation and G×E interaction modeling. A more detailed genotype-level analysis such as identifying the most influential genetic markers could provide additional biological interpretability.

## 6 Conclusion

This paper proposed a GNN+LSTM joint model for G×E yield prediction. The GNN is responsible for capturing non-linear genotype-environment interactions through message passing, and the LSTM is responsible for transforming raw weather time series into compact environment embeddings. Two critical design approaches were local links (among genotypes and among environments) and a global connection (the supernode readout after message passing). The combination of these improved correlation and calibration.

Future work may consider alternative representations of genomic information. In the current framework, genotype data are compressed using PCA, and each principal component is treated as a node in the graph. Although this reduces dimensionality, it may remove some marker-level structure [41]. An alternative approach would be to use the original marker sequences directly or to construct genotype-level nodes that preserve more detailed genetic information.

In addition, the present graph represents genomic components and environmental features as shared nodes across all samples. It could be beneficial to define each node as a specific genotype or environment instance rather than as feature components. Edges could capture G×E interactions and model their relationships more explicitly. This sample-level graph structure may provide a more direct representation of biological entities and interaction patterns.

Furthermore, the PCA projection of the test environments showed that most environments were concentrated near the center, with no clear performance differences in more extreme regions. This observation suggests that the current framework may primarily capture average environmental patterns. Future studies may therefore explore incorporating additional strategies to better account for extreme climatic conditions.

## Supporting information

S1 Appendix Appendix. (PDF)
