# Supplementary material for: LSTM-attention-guided graph neural networks for integrated genotype–Environment modeling in maize yield prediction
Source: PLoS Comput Biol. 2026 May 20;22(5):e1013729. doi: 10.1371/journal.pcbi.1013729 (PMC13215614; doi:10.1371/journal.pcbi.1013729)
Supplement: S1 Appendix — (PDF) [file pcbi.1013729.s001.pdf]

S1 Appendix

Environmental feature selection using XGBoost and SHAP

To identify the most important environmental variables, an XGBoost model was trained as a classifier, and SHAP values were computed to quantify feature importance. For model training, each unique environment in the weather dataset was treated as a single observation. For every environment, the average value of each environmental feature over the corresponding time period was calculated. The average yield for each environment, obtained from the trait file, was used as the target variable.

The trained XGBoost model was subsequently interpreted using SHAP. SHAP values range from negative to positive contributions, where values closer to $+1$ indicate a strong positive contribution of a feature to the prediction, and values closer to -1 indicate a strong negative contribution. Fig A illustrates the overall procedure used for environmental feature selection.

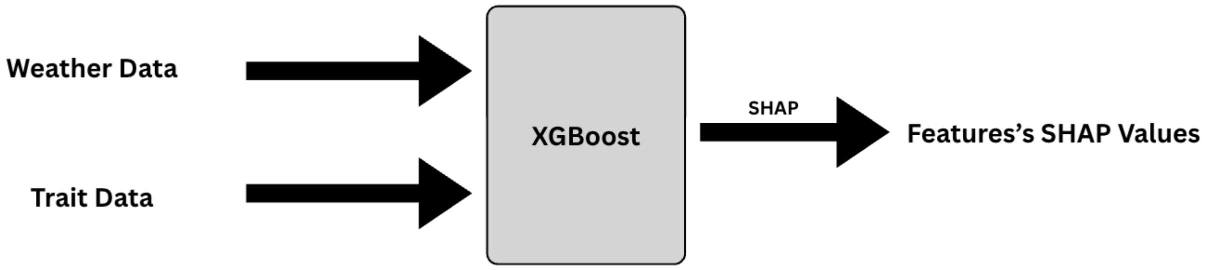

Figure A: Workflow for environmental feature importance analysis.

SHAP values were calculated for all 16 environmental features to find their contributions to yield prediction. The SHAP values are summarized in Table A. Four features were selected from the top-ranked variables for model development. `T2M_MIN` was included together with `T2M_MAX` to better represent temperature variability, which may influence plant development beyond peak temperature effects alone.

Future work may explore alternative feature selection strategies beyond SHAP-based ranking alone.

Table A: SHAP values for all environmental features.

Feature	SHAP Value
ALLSKY_SFC_SW_DNI	0.628218
WS2M	0.478706
ALLSKY_SFC_PAR_TOT	0.296091
PS	0.119694
ALLSKY_SFC_SW_DWN	0.111621
T2M_MAX	0.092846
PRECTOTCORR	0.089835
GWETTOP	0.084046
QV2M	0.078980
GWETPROF	0.071405
RH2M	0.067707
GWETROOT	0.064419
T2M	0.056051
T2MDEW	0.051417
T2M_MIN	0.042878
T2MWET	0.038520

Genotype PCA explained variance

Fig B shows the cumulative explained variance of the genotype PCA. The selected 548 principal components retain 87.57% of the total variance in the filtered SNP matrix, supporting the use of PCA as a compact representation of genomic information in the proposed framework.

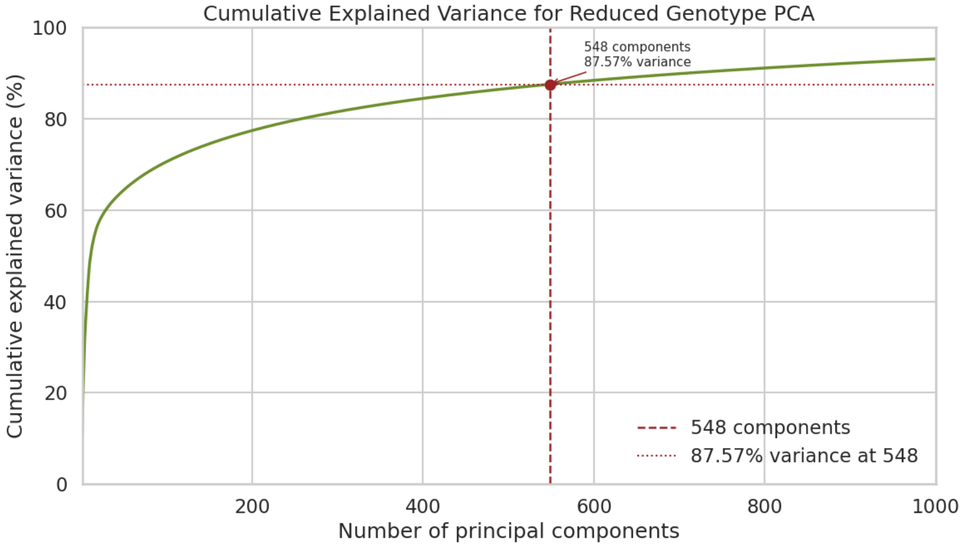

Figure B: Cumulative explained variance of the genotype PCA. The selected 548 principal components retain 87.57% of the total variance in the filtered SNP matrix.
